# Activation of the NF**κ**B Pathway Enhances AhR Expression in Intestinal Caco-2 Cells

**DOI:** 10.1155/2013/792452

**Published:** 2013-10-21

**Authors:** S. Champion, C. Sauzet, P. Bremond, K. Benbrahim, J. Abraldes, E. Seree, Y. Barra, P. H. Villard

**Affiliations:** ^1^IMBE-UMR CNRS 7263, IRD 237 Aix-Marseille Université Campus Timone, Faculté de Pharmacie, 27 boulevard Jean Moulin, 13385 Marseille Cedex 05, France; ^2^UMR INSERM 1062, INRA 1260, Nutrition, Obésité et Risque Thrombotique (NORT), Aix-Marseille Université Campus Timone, Faculté de Pharmacie, 27 boulevard Jean Moulin, 13385 Marseille Cedex 05, France; ^3^Laboratoire de Génie Génétique, Aix-Marseille Université Campus Timone, Faculté de Pharmacie, 27 boulevard Jean Moulin, 13385 Marseille Cedex 05, France

## Abstract

Recent data suggest that apart from its well-known role in the regulation of xenobiotic metabolizing enzymes, AhR is also involved in inflammation. However, the influence of inflammation on AhR expression remains unknown. Here, we demonstrated that proinflammatory conditions induced by either PMA or IL-1**β** enhance AhR expression in Caco-2 cells. This was associated with an increase in AhR promoter activity. By means of directed mutagenesis experiments and the use of proteasome inhibitors, we demonstrated that inflammation-induced AhR expression involved the NF**κ**B pathway but not AP-1. Moreover, conditioned media from PMA-treated Caco-2 cells were also able to induce AhR expression, and this induction was repressed by anti-IL-1**β** blocking antibodies. Similar results were obtained with conditioned media from PMA-treated THP-1 cells. Taken together, these data suggest that AhR could be involved *in vivo* in an inflammatory loop. AhR was recently suspected to be implicated in inflammatory bowel disease. Our results support this hypothesis and suggest that AhR could be a new target for inflammatory bowel disease patient management.

## 1. Introduction

The aryl hydrocarbon receptor (AhR) is a transcription factor activated by numerous environmental ligands such as dioxins and polycyclic aromatic hydrocarbons (PAHs) [1]. Its endogenous ligand has not yet been described, but some endogenous compounds, notably oxidative derivatives of tryptophan, are already described as efficient activators. Following ligand binding, AhR translocates to the nucleus, dimerizes with its partner the aryl hydrocarbon receptor nuclear translocator (ARNT), and binds to xenobiotic responsive elements (XRE) in target genes.

AhR is known to be a key regulator of some xenobiotic degradation enzymes, notably cytochromes P450 belonging to the CYP1 family, which are involved in the bioactivation of various environmental procarcinogens including PAH and arylamines. The AhR-mediated pathway is commonly viewed as an “adaptive” response toward these xenobiotic agents. 

Recent data demonstrated that AhR mediates diverse endogenous functions in our close vertebrate relatives as well as our distant invertebrate ancestors, including cell proliferation, adhesion and migration, and inflammation [2, 3]. Accidental exposure to dioxins, which are prototypes of environmental AhR ligands, leads to a broad spectrum of pathologies, ranging from cancers to cardiovascular diseases and type 2 diabetes [4–6], all of which involve an inflammatory process. Using a “triple-null” mouse model that lacks the two receptors for TNF*α* and TNF*β* and the receptor for the IL-1*α* and IL-1*β* cytokines, it was demonstrated that IL1-like cytokines play a central role in dioxin-induced inflammatory effects [7]. We have shown in intestine that PAH-induced AhR activation upregulates the expression of some inflammation target proteins, including proinflammatory cytokines such as IL-1*β* and TNF*α* [8, 9]. Similar data have been observed in other cells and tissues, ranging from macrophages and breast cells to skin and lung [10–13]. Moreover, Hollingshead et al. showed that 2,3,7,8-tetrachlorodibenzo-p-dioxin (TCDD) treatment in combination with IL-1*β* or phorbol 12-myristate 13-acetate (PMA) results in a marked synergistic induction of IL-6 levels over what is seen without AhR activation [11]. Since TCDD induces IL-6 expression through the AhR pathway, this synergistic effect could be partly explained by an inflammation-induced increase in AhR expression.

The aim of this study on Caco-2 cells was to investigate the effect of signals known to be proinflammatory on AhR expression and to describe the molecular mechanisms involved.

## 2. Materials and Methods

### 2.1. Chemicals and Reagents

Phorbol 12-myristate 13-acetate (PMA) was sourced from Sigma (France), IL-1*β* from Peprotech (France), anti-IL1*β* antibody (ab2105) from Abcam (France), and Proteasome Inhibitor Set I from Calbiochem (France).

### 2.2. Culture and Cell Treatments

CaCo-2 human colonic adenocarcinoma cells and THP1 human monocytic cells were cultured as previously described [8, 14]. At confluence, cells were starved for 12 h without FBS (replaced by 0.2% BSA) and treated for 1 h to 24 h with either 100 nM PMA or 200 nM IL-1*β*.

In some experiments, Caco-2 or THP-1 cells were treated with conditioned media. To obtain the conditioned media, cells were treated for 2 h with 100 nM PMA, washed 3 times with PBS, and further cultured in 0.2% BSA medium. Media samples were collected after 2–24 h incubation, and a new Caco-2 batch was treated for 8 h with these conditioned media.

### 2.3. Quantitative RT-PCR Experiments

Total RNA was isolated using a Nucleospin RNAII kit (Macherey-Nagel, France) and reverse-transcribed at 42°C for 1 h using GibcoBRL M-MLV reverse-transcriptase (Life Technologies, France) and random primers.

Expression levels of target genes (AhR, IL1-*β*, IL-8, TNF*α*, and TGF*β*) were determined using a LightCycler 480 System (Roche, France). PCR was performed with 0.5 *μ*M of each primer using the LightCycler with Mastermix Plus for SYBR Green I No ROX. Cycling conditions were 10 min denaturation at 95°C, followed by 40 cycles of 30 s denaturation at 95°C, 30 s primer annealing at 60°C, and 30 s fragment elongation at 72°C. The melting curve was analyzed on LightCycler 480 gene scanning software. AhR, IL-1*β*, IL-8, TNF*α*, and TGF*β* mRNA expressions were normalized to *β*2-actin expression, and data were quantified by the 2^−ΔΔCt^ method. The primers used are listed in Table 1.

### 2.4. Ahr Promoter Luciferase Assays

The 2.7 kb of the human AhR gene 5′-flanking region (the −2103/+637 region of the AhR gene) was subcloned into the pGL3-enhancer luciferase vector (Promega, France) as previously described [8] to obtain the p3.48 construct.

Caco-2 cells in six-well plates were grown to 50–60% confluence before transfection. Transient transfections were performed by lipofection (lipofectin, Life Technologies) in a serum-free and antibiotic-free medium containing 2% L-glutamine, with 0.5 *μ*g of p3.48. After 48 h treatment with 100 nM PMA, luciferase activity was evaluated using the Luciferase Assay System from Promega. 

### 2.5. Site-Directed Mutagenesis of AP1 and NF*κ*B Sites of the AhR Promoter

AhR promoter analysis with Mathinspector software (Genomatix Software, Germany) revealed the presence of 3 AP1 and 3 NF*κ*B putative binding sites. These sites were mutated using the QuickChange site-directed mutagenesis kit (Stratagene, France). Sequences of sense primers used for mutagenesis are listed in Table 2. Presence of the mutations was checked by restriction analysis and verified by DNA sequencing.

Cells were transfected with 0.5 *μ*g of the mutated vectors, and after a 48 h treatment with 100 nM PMA, luciferase activity was evaluated as described above.

### 2.6. Statistical Analysis

Statistical analysis was performed using a Mann-Whitney test on GraphPad Prism (GraphPad Software). Values were considered statistically different at *P* < 0.05. Results are presented as means ± SD. 

## 3. Results

### 3.1. Effect of PMA or IL-1*β* Treatments on AhR Transcript Levels

In order to evaluate the effect of proinflammatory conditions on AhR mRNA levels, Caco-2 cells were treated with PMA or with IL-1*β*.

**Figure 1:**
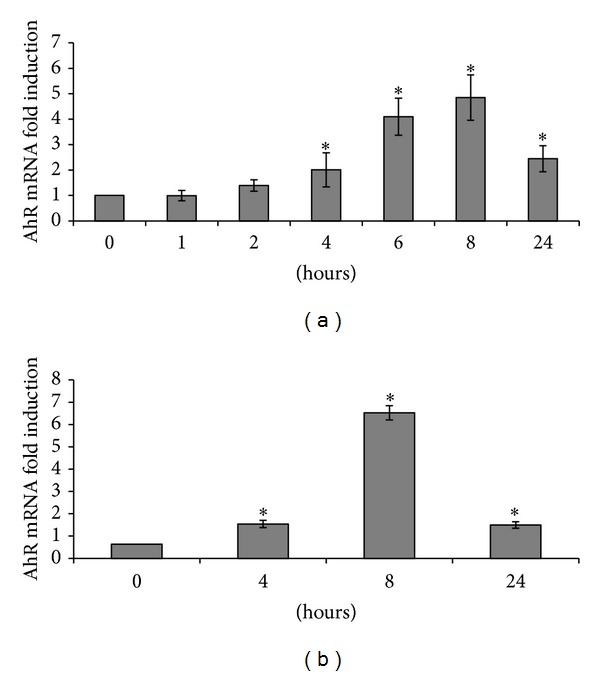
Effects of 100 nM PMA (a) and 200 nM IL-1*β* (b) on AhR mRNA levels. *: *P* < 0.05* versus* control.

**Figure 2:**
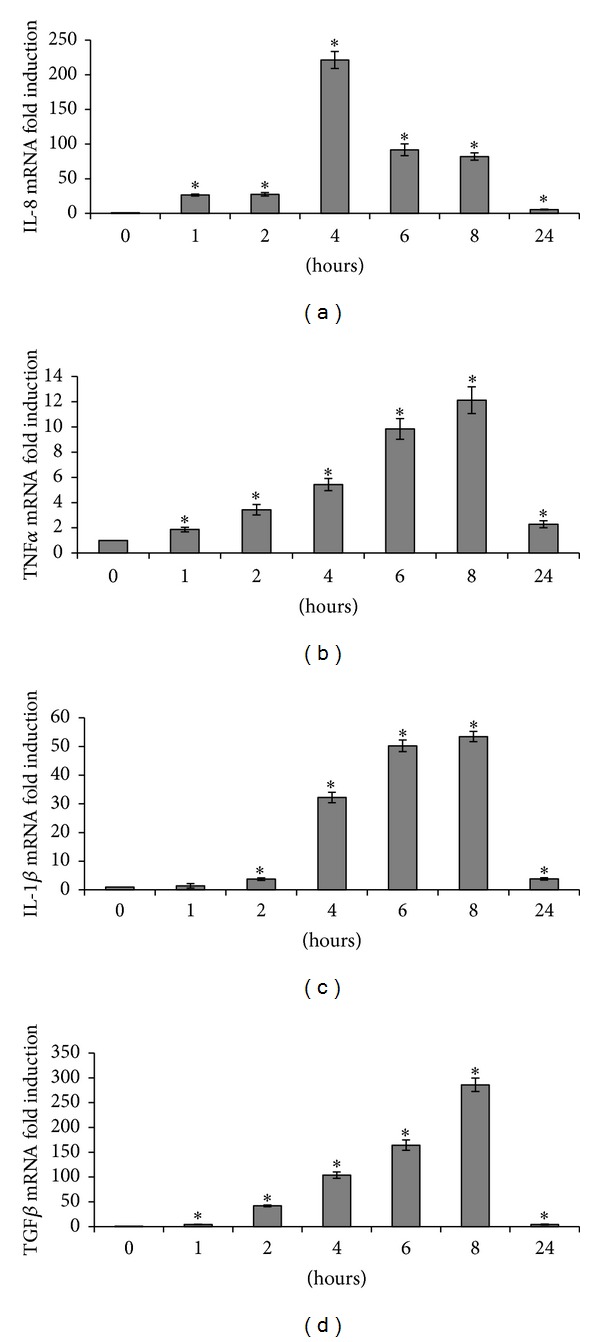
Effect of 100 nM PMA on IL-8 (a), TNF*α* (b), IL-1*β* (c), and TGF*β* (d) mRNA levels. *: *P* < 0.05* versus* control.

The maximal (4.9-fold) induction of AhR mRNA was observed after 8 h of treatment with PMA (Figure 1(a)). We also evaluated the expression of various cytokines after exposure to PMA (Figure 2). Peak IL-8 upregulation (92-fold) occurred after 4 h of exposure. Peak TNF*α*, IL-1*β*, and TGF*β* upregulation (10-, 53-, and 286-fold, resp.) occurred after 8 h of exposure.

Treatment of Caco-2 cells with the proinflammatory cytokine IL-1*β* was also associated with an increase in AhR mRNA that was maximal (6.5-fold) after 8 h of treatment (Figure 1(b)).

Taken together, these results showed that enhancement of AhR expression was associated with signals involved in proinflammatory processes.

### 3.2. Effect of PMA Treatment on Activation of the AhR Promoter

**Figure 3:**
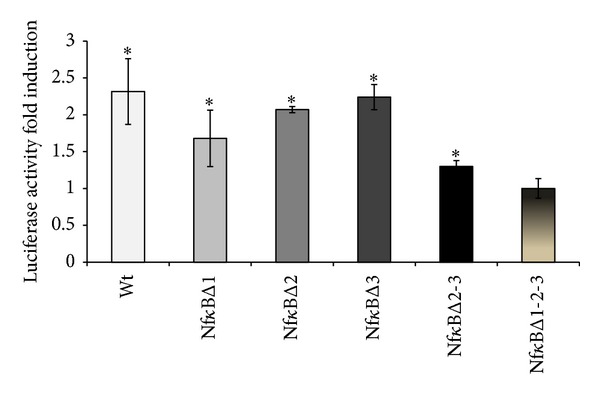
Effect of 100 nM PMA on AhR promoter activity after sequential mutation of the 3 putative NF*κ*B binding sites. *: *P* < 0.05* versus* untreated cells.

To see whether AhR induction (mRNA) in response to PMA was associated with an increase in AhR transcription, reporter gene expression was analyzed using the p3.48 construct in which luciferase expression was driven by the AhR promoter. Treating WT p3.48-transfected Caco-2 cells with 100 nM PMA led to a 2.3-fold increase in luciferase expression (Figure 3), showing that increased AhR expression in response to PMA was mainly of transcriptional origin.

PMA is well known to potentialize inflammation-related processes through AP-1 and NF*κ*B pathways. AhR promoter analysis using Matinspector software revealed the putative presence of 3 AP-1 and 3 NF*κ*B binding sites. The mutation of AP-1 sites did not modify AhR induction by PMA (data not shown), suggesting that only the NF*κ*B pathway was involved. The effects of mutations of the three NF*κ*B binding sites found in the AhR promoter are summarized in Figure 3. The mutation of one of the three sites did not significantly modify luciferase induction, whereas mutation of the first site proved most efficient. Double mutation of sites 2 and 3 reduced the induction of luciferase expression by 45%, while mutation of all three sites totally abrogated this induction. Taken together, these data strongly suggest that AhR induction involves the NF*κ*B pathway.

### 3.3. Effect of a Proteasome Inhibitor Cocktail on AhR and IL-1*β* mRNA Induction by PMA

**Figure 4:**
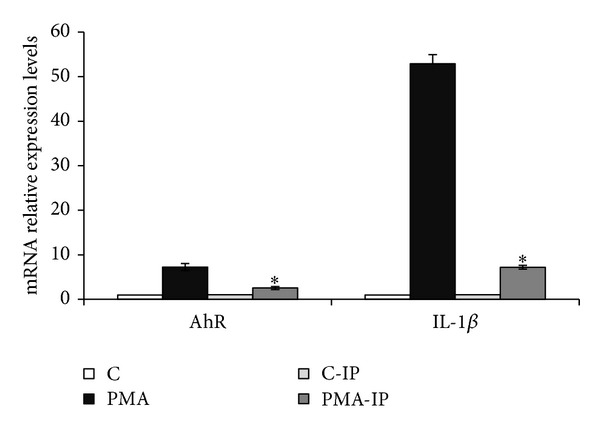
Effect of proteasome inhibitor cocktail on 100 nM PMA-mediated AhR and IL-1*β* transcript induction. C: control Caco-2 cells; PMA: 100 nM PM-treated Caco-2 cells; C-IP: control Caco-2 cells pretreated with proteasome inhibitor cocktail; PMA-IP: Caco-2 cells pretreated with proteasome inhibitor cocktail and treated with 100 nM PMA. *: *P* < 0.05* versus* control.

In order to gain stronger confirmation of the role of NF*κ*B in AhR expression, we reduced NF*κ*B activation by inhibiting I*κ*B degradation using a supplier-specified proteasome inhibitors cocktail that includes proteasome inhibitor I, lactacystin, and MG-132. As shown in Figure 4, using the proteasome inhibitor cocktail led to a 65% reduction in AhR induction by 100 nM PMA, along with an 86% decrease in IL-1*β* enhancement, demonstrating that the proteasome inhibitor cocktail was able to prevent the IL-1*β* induction triggered by the NF*κ*B transduction pathway.

### 3.4. Effect of Conditioned Media from PMA-Treated Caco-2 Cells or from PMA-Treated THP-1 Cells on AhR Expression

**Figure 5:**
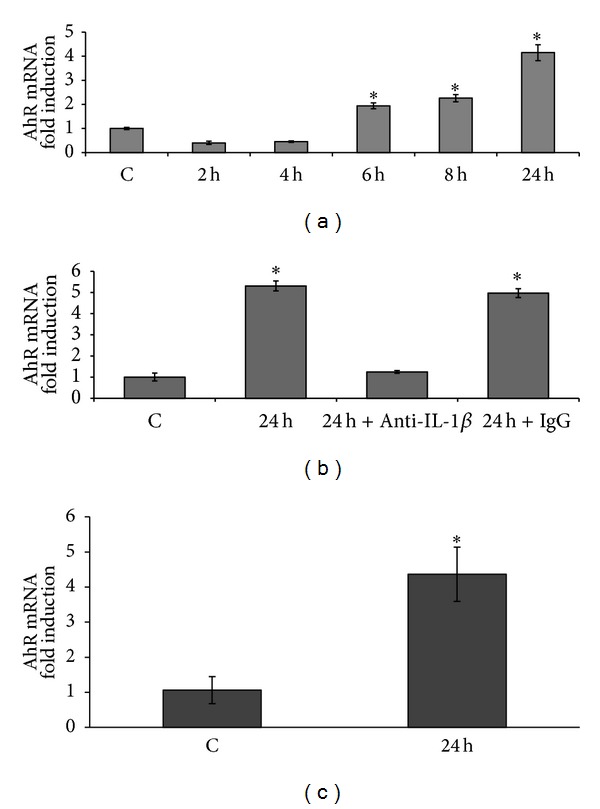
Effect of 8 h exposure to conditioned media from 100 nM PMA-treated Caco-2 cells on AhR mRNA levels (a). Effect of 8 h exposure to conditioned media from 100 nM PMA 24 h treated Caco-2 cells in presence of IL-1*β*-neutralizing antibodies on AhR mRNA levels (b). Effect of 8 h exposure to conditioned media from 100 nM PMA-treated THP-1 cells on AhR mRNA levels (c). *: *P* < 0.05* versus* control.

Caco-2 cells are able to produce cytokines, notably TNF*α* and IL-1*β*, in response to proinflammatory signals [15]. These cytokines exert their effect through the NF*κ*B pathway. Therefore, treating Caco-2 cells with conditioned media from PMA-treated Caco-2 cells should result in AhR induction. Our results are summarized in Figure 5. Media collected from 6 to 24 h after treating Caco-2 cells with PMA significantly upregulated AhR mRNA. Maximal activity (4.2-fold increase) was obtained with the 24 h conditioned medium (Figure 5(a)). Pretreating the cells with an IL-1*β* neutralizing antibody (dilution 1/100) 4 h before exposure to 24 h PMA-conditioned medium inhibited the induction of AhR expression, while pretreatment with rabbit isotype IgG had no effect (Figure 5(b)). In another experiment, Caco-2 cells were treated with conditioned media from PMA-treated THP-1 cells, and similarly we observed an induction of AhR mRNA (4.4-fold increase) (Figure 5(c)). Our data therefore point to the involvement of a signalization loop which could lead to an enhancement of inflammatory processes.

## 4. Discussion

AhR activation is known to induce proinflammatory cytokine expression. This study suggested the induction of an inflammation loop resulting from an initial AhR activation in the colon. Indeed, this tissue through diet is effectively chronically exposed to various AhR ligands such as PAH or food residues like dioxins or polychlorobiphenyls.

Our results obtained in Caco-2 cells clearly demonstrated that both PMA- and IL1-*β* enhance AhR transcript expression. This phenomenon was associated with an increase of AhR promoter activity. As inflammation-related processes mainly involve NF*κ*B and AP-1 transduction pathways, we carried out site-directed mutagenesis of AP-1 or NF*κ*B binding sites. Mutagenesis of AP-1 was unable to decrease the induction of AhR promoter activity, whereas mutation of the 3 putative NF*κ*B binding sites abrogated the increase in AhR promoter activity. We also pretreated cells with a proteasome inhibitor cocktail in order to prevent degradation of the I*κ*B subunit and therefore inhibit NF*κ*B activation. This pretreatment inhibited both the induction of AhR expression after PMA exposure and the increase of IL-1*β* expression, which is known to be mainly regulated by NF*κ*B. Taken together, these results demonstrated that proinflammatory conditions induce AhR expression at least partly through the NF*κ*B pathway.

Caco-2 cells express a number of cytokine receptors on their cellular membrane and are also able to secrete proinflammatory cytokines in response to initial inflammatory signals. These cytokines exert some of their effect through the activation of NF*κ*B. Our results demonstrated that treating Caco-2 cells with a conditioned media derived from PMA-treated cells also leads to an increase of AhR expression, and that this induction involved IL-1*β* signaling. Similar results were obtained with human monocytic THP1 cells. These results are consistent with our results from site-directed mutagenesis experiments and the treatments of cells with proteasome inhibitors. Moreover, these data suggested that an autocrine loop could occur and probably generate and amplify a proinflammatory signal. Indeed, environmental exposure to AhR agonists like PAHs has been demonstrated to induce the expression of proinflammatory cytokines such as IL-1*β* [8, 9] and to activate NF*κ*B [16]. Our data demonstrated that proinflammatory conditions induced and sustained through AhR expression could, therefore, increase cell susceptibility to PAH-induced inflammation.

**Figure 6:**
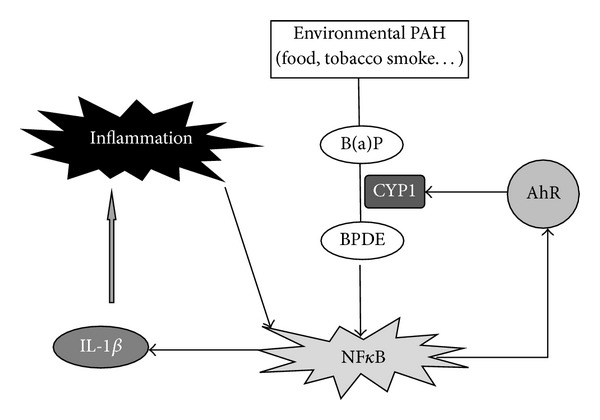
Hypothetical role of the AhR pathway in the development of inflammatory bowel disease after exposure to environmental PAH. B(a)P: benzo(a)pyrene; BPDE: benzo(a)pyrene diol epoxide.

The environment exposes us to various AhR ligands that could modulate susceptibility to inflammation. PAHs are potent AhR ligands that are present in tobacco smoke as well as diet, notably grilled meats. Cigarette smoking is emerging as a strong risk factor in the otherwise unknown etiology of chronic inflammatory diseases [17]. There are reports of a dose-response relationship between exposure to tobacco smoke and inflammatory bowel disease (IBD) [18]. The exact mechanisms by which smoking influences the development of IBD are poorly understood, but nicotine does not appear to play a critical role [18]. Interestingly, a recent study in dextran sulfate sodium-induced colitis mice reported that the attenuation of AhR expression resulted in a protective effect [19]. Moreover, AhR and its downstream targets, such as IL-8, were significantly upregulated in IBD patients versus controls. The authors concluded that abnormal AhR pathway activation in the intestinal mucosa of IBD patients may promote chronic inflammation [19], and our results support this hypothesis.  Figure 6 proposes a possible explanation of the link between the AhR pathway and IBD. PAHs such as benzo(a)pyrene, are bioactivated by CYP1 family enzymes into diolepoxides, such as benzo(a)pyrene diol epoxide [20], which activate NF*κ*B [16]. The activation of NF*κ*B promotes an inflammatory loop via IL-1*β* expression and induces AhR expression. The upregulation of AhR would, in response to PAH exposure, enhance both CYP1 inducibility and PAH-inflammatory properties.

NF*κ*B-controlled pathways were classically divided into two branches: the classical pathway involving RelA subunit and IKK*β* and the alternative pathway involving RelB subunit and IKK*α*. Physical interactions between AhR and each one of the NF*κ*B subunits were reported to induce distinct effects via specific sequences. Interaction of AHR with RelA induced a downregulation of gene expression controlled by RelA as in the case of CYP1A1 [21] or IL-6 gene [22]. In opposite, interaction of AhR with RelB enhances DRE-reporter gene activity of CYP1A1 and transcription of some NF*κ*B target genes such as IL-8 and other chemokines through binding on specific RelB/AhRE sequences [23, 24]. Furthermore, NF*κ*B-binding sites that are preferentially recognized by RelB/p52 are spontaneous targets for RelB/AhR complexes (i.e., independently of addition of any exogenous ligand). RelB/AhR complexes are also found to bind on XRE, as well as NF*κ*B consensus elements, and RelB drastically increases the TCDD-induced XRE-Luc reporter activity. Vogel and Matsumura [25] propose that AhR assists the function of RelB not only to mediate chronic inflammation but also to promote RelB's function in resolution of inflammation via negative feedback mechanisms, whereas AhR antagonizes the action of RelA to moderate acute cellular inflammation and/or protect cells from unwanted side effects of full activation of inflammatory effects of RelA. Our data showed that *in vitro*, proinflammatory conditions enhance AhR expression through NF*κ*B pathway, therefore, it would be of interest to evaluate if the enhanced expression of AhR was also associated with an increase of RelB/AhR complex formation and if such an interaction promotes *in vivo *either inflammation or its resolution. 

In conclusion, we demonstrated for the first time that compounds inducing proinflammatory cytokine expression enhance AhR expression in intestinal epithelial Caco-2 cells through the NF*κ*B transduction pathway. Several pieces of evidence point to AhR as a potential new target in the management of IBD and suggest that the modulation of AhR signaling pathway via diet, smoking cessation, or the consumption of AhR antagonists such as resveratrol [26] could be a viable new strategy for the prevention and treatment of IBD.

## Figures and Tables

**Table 1:** Sequences of primers used in qRT-PCR experiments.

Primers	Sequence
*β* actin-F	5′ CCCAGCACAATGAAGATCAA 3′
*β* actin-R	5′ CGATCCACACGGAGTACTTG 3′
AhR-F	5′ CAGAAAACAGTAAAGCCAATCC 3′
AhR-R	5′ AATACAAAGCCATTCAGAGCC 3′
IL1*β*-F	5′ AACAGGCTGCTCTGGGATT 3′
IL1*β*-R	5′ TGGCTGCTTCAGACACTTGA 3′
IL8-F	5′ AGACAGCAGAGCACACAAGC 3′
IL8-R	5′ ATGGTTCCTTCCGGTGGT 3′
TNF*α*-F	5′ CAGCCTCTTCTCCTTCCTGA 3′
TNF*α*-R	5′ GCCAGAGGGCTGATTAGAGA 3′
TGF*β*-F	5′ CCGGATACTCAGGCCAGA 3′
TGF*β*-R	5′ AGAGATACGCAGGTGCAGGT 3′

**Table 2:** Sequences of primers used insite-directed mutagenesis experiments.

Sense primers (location)	Sequence
AhR_AP1-Mut1_ (−626/−578)	5′ CTGCATTCACGAAAGTCATC**AGCTACTACAC**ATTGAGAAAACAAGAATG 3′
AhR_AP1-Mut2_ (−1125/−1077)	5′ GCTCCTCCAACTTTATGTACA**TTCAAATAACC**TGGGAGTTCCTGTGAAC 3′
AhR_AP1-Mut3_ (−1526/−1477)	5′ GATTCTGCCTC**TGCAATGGCTAAGGTATAAACATCA**AACTTTCCCAGTG 3′
AhR_NFκB-Mut1_ (−432/−382)	5′ CCCGCACACCAAAAAAGGT**CAAGGTACCTCCTAG**CCTTCAAGTCTCAACTC 3′
AhR_NFκB-Mut2_ (−1115/−1066)	5′ CTTTATGTACATTTGAATCA**CCTGGTACCCCTGT**GAACTTCGGGTTCTG 3′
AhR_NFκB-Mut3_ (−1482/−1432)	5′ CAGTGTACACTGTCTTCT**TTGGTACCTTGCTCC**ATCTTTTTCCTTAAACTG 3′

Binding site sequences are in bold, and mutated bases are underlined.
